# Aflatoxin exposure in children age 6–12 years: a study protocol of a randomized comparative cross-sectional study in Kenya, East Africa

**DOI:** 10.1186/s40814-019-0510-x

**Published:** 2019-11-29

**Authors:** Ruth Nabwire Wangia, David Peter Githanga, Jia-Sheng Wang, Omu Aggrey Anzala

**Affiliations:** 1 Department of Environmental Health Science, College of Public Health, University of Georgia, Athens, GA 30602 USA; 2 KAVI Institute of Clinical Research, University of Nairobi, P.O. Box 19676–00202, Nairobi, Kenya; 3 Department of Medical Microbiology, University of Nairobi, P.O. Box 19676–00202, Nairobi, Kenya

**Keywords:** Aflatoxins, Children, Adverse health outcomes, Immune suppression, Growth impairments

## Abstract

**Background:**

Aflatoxins (AFs) are naturally occurring fungal metabolites produced by the *Aspergilla* species of fungi. The staple food grain, maize (*Zea mays*), is highly susceptible to AF contamination. In Kenya, contamination of maize supplies by AFs is a recognized public health problem which has resulted in over 600 human deaths. Human exposure to AFs can occur in utero, via breast milk, through weaning foods, and throughout an individual’s lifetime. Recent epidemiological studies have shown that exposure to AFs in early life through diet is a contributing factor to immune suppression, micronutrient deficiency, possible vaccine interference, and impaired growth in children. However, these results remain inconsistent and inconclusive due to lack of randomized controlled studies.

**Methods:**

A randomized school-based cross-sectional study was designed to study AF exposure levels and associated health effects in children between ages 6 and 12 years. Participants were recruited from primary schools within Siaya and Makueni Counties of Kenya, East Africa. The Joint Ethics Committee of the University of Nairobi and Kenyatta National Hospital in Kenya approved the research protocol and procedures for the study. Both parental consent and child assent were obtained before enrollment in the study. Parents were requested to provide household grain samples and fill out questionnaires detailing their sociodemographic information, household dietary patterns, farming practices, and knowledge of AF contamination. Blood samples were collected from children participants, and sera were prepared for analysis of AFB_1_-lysine which is one of the validated biomarkers for AF exposure.

**Discussion:**

This protocol describes a school-based, cross-sectional study whose objective is to comparatively evaluate the role of AF exposure on adverse health outcomes in children. Specifically, effects of cumulative AF exposure on nutritional status, immune markers, and growth parameters will be assessed.

**Trial registration:**

This study is not a clinical trial, rather a cross-sectional study aimed at providing baseline data on AF exposures in children who live in presumably high versus low AF exposure regions. Results from the study can be used to design interventions and/or prospective cohort studies aimed at studying adverse health effects associated with cumulative AF exposure through diets. The study reference number is P741/12/2017 and registered with KNH-UoN Ethics and Research Committee.

## Background

Aflatoxins (AFs) are a group of naturally occurring mycotoxins produced by the common fungus *Aspergillus flavus* and the closely related *Aspergillus parasiticus* fungi [1–3]. Up to 4.5 billion people around the world are exposed to AFs through the diet and/or via occupational exposures during grain handling [4, 5]. AFs are common food contaminants which present a persistent challenge throughout the food chain. Food staples frequently contaminated with AFs include maize, peanuts, rice, cassava, spices, and other food items [1–3].

There are up to 14 different groups of naturally occurring AFs, but the commonly studied groups include B_1_, B_2_, G_1_, and G_2_ [1, 3]. Aflatoxin B_1_ (AFB_1_) is the most biologically potent because it is a confirmed human carcinogen classified into group I by the International Agency for Research on Cancer [2, 3]. AFB_1_ is a major risk factor associated with primary liver cancers as evident in many studies conducted in Africa and South East Asia [6–8]. Moreover, AFs are immune toxicants and have been associated with immune suppression in human populations [9, 10]. In children, recent epidemiology studies provide evidence that cumulative exposure to AFs in low concentrations contributes to micronutrient deficiency, possible vaccine interference, immune suppression, and growth impairments [11–13]. The adverse health outcomes associated with AF exposure may persist into adulthood if neither interventions nor corrective measures are undertaken. To date, comprehensive data on AF exposure is limited, and thus, assessment of adverse health outcomes is further hindered.

Contrary to long-term exposure to AFs where adverse health outcomes occur over time, dietary exposure to AFs exceeding 200 μg/kg in the short term can be fatal due to aflatoxicosis [14–16]. Aflatoxicosis is a medical condition characterized by jaundice, bile duct proliferation, edema, sudden liver failure, and ultimately death within 24 h of consumption of AF-contaminated maize [15–18].

In Kenya, AF contamination in maize supplies is a recognized public health problem, which has resulted in more than 600 documented human deaths attributed to aflatoxicosis [19–21]. The US Food and Drug Administration (USFDA) recommends that food destined for human consumption should not exceed total AFs of 20 μg/kg [22]. The European Union’s Codex Alimentarius recommends 15 μg/kg [23] while Kenya, given its troubled past with deaths associated with aflatoxicosis, stipulated a strict recommendation of 10 μg/kg [24]. These recommended exposure levels are significantly difficult to implement due to widespread subsistence farming which promotes higher AF exposure because maize produced in farms are consumed directly without prior testing for AF contamination levels.

### Study hypotheses, objective, and specific aims

We hypothesize that children recruited from high AF exposure region of Makueni are more likely to suffer adverse health outcomes including micronutrient deficiency, immune suppression, and growth impairment compared to children recruited from low exposure region of Siaya County. This hypothesis will be tested against the null hypothesis of dietary exposure to AFs is not associated with adverse health outcomes in children.

The general objective of the study is to provide a comprehensive assessment of how dietary exposure to AFs contributes to micronutrient deficiency, immune suppression, and growth impairment in school-going children. Specific aims include the following: (1) to determine the cumulative levels of AF exposure in children of Makueni and Siaya Counties; (2) to determine the nutritional and immune status of children in Siaya and Makueni Counties; (3) to study the association between AF exposure, micronutrient deficiency, and immune suppression; and (4) to elucidate possible growth impairment, if any, in children with high AFB_1_-lysine adducts exceeding 10 pg/mg of albumin.

This study will be the first to provide baseline data on AF exposure in children between the ages of 6 and 12 years recruited from a high and low AF exposure regions of Kenya. The study is a collaborative project between the University of Nairobi’s KAVI Institute of Clinical Research and the University of Georgia in Athens in the USA. The study protocol is based on the SPIRIT (Standard Protocol Items: Recommendations for Interventional Trials) and CONSORT 2010 (Consolidated Standards of Reporting Trials) reporting guidelines.

## Methods/design

The study design is school-based and cross-sectional to provide a snapshot of AF exposure levels among children recruited from Siaya and Makueni Counties. Cross-sectional study design was preferred due to its ability to generate factual information on AF exposure among children widespread over two geographic locations. To ensure samples are representative of both Makueni and Siaya Counties, primary schools without feeding programs were randomly selected in different constituencies per county.

### Setting of the study

The selection of Makueni and Siaya Counties was made according to previous studies which reported high AF exposures in Makueni County while Siaya’s exposure levels were below the level of detection [15, 25]. Makueni County lies in Kenya’s former Eastern Province while Siaya County forms one of the six counties in the former Nyanza Province [25]. Siaya County is hot and humid with temperatures averaging from 21 to 25 °C while annual precipitation ranges between 1000 and 1750 mm [26]. Conversely, the climate of Makueni County is predominantly semi-arid characterized by long dry seasons interspersed by an annual rainfall of about 500 mm and ambient temperature ranges between 18 and 24 °C in the cold season and between 24 and 33 °C in the hot season [27]. Makueni County’s aridity has been implicated to be a significant contributing factor to AF contamination as a result of undue crop stress [15, 27]. The Luo ethnic groups are the main inhabitants of Siaya County while the Akamba community inhabits the high exposure regions of Makueni County [28]. The estimated poverty rate in Makueni and Siaya Counties is 64% and 48%, respectively, compared to the national average of 47% as of 2009 [26, 28]. Overall, the choice of Makueni and Siaya Counties as study location provides control for differences in weather conditions, ethnicity, and poverty levels.

Due to limited food diversity in sub-Saharan Africa, our study populations depend on maize and maize-based products for daily energy requirements [29, 30]. The dietary staples in Kenya include maize-based meals comprised of solid maize meal, commonly known as *ugali* in Swahili; porridge; roasted and boiled maize cobs; and/or maize boiled with beans [30]. Moreover, each household is estimated to consume maize-based meals at least two times a day because *ugali* is often served during lunch and/or dinner in accompaniment with a mix of vegetables and/or any kind of stew [30, 31]. Consuming a variety of foods from several food groups is a recommended approach to achieve necessary nutritional requirements and limit excessive exposure to AFs through the diet [32]. Diet diversification has been proposed to be instrumental in mitigating AF exposure and the associated adverse health outcomes. The International Food Policy Research Institute proposed that households with a dietary score less than 4.5 can be categorized as low dietary diversity, a score between 4.5 and 6 has medium dietary diversity, and a score greater than 6 can be considered both high and good dietary diversity [33]. In our study populations, past studies reported low dietary diversity with a score of less than 4 food groups each day [31, 34]. Therefore, food insecurity is paramount in our study populations due to low agricultural productivity that is characterized by unreliable rains, limited access to farm inputs, and hired labor which increases the cost of food production significantly [26]. Nonetheless, small-scale holder farmers undertake maize cultivation for household consumption despite low agricultural output and increased risk of AF contamination.

### Justification of Makueni and Siaya Counties as study sites

In Kenya’s former Eastern Province, high level of AF contamination is concentrated in Makueni County as evident in multiple aflatoxicosis outbreaks previously reported [15, 18]. In 2004, a severe aflatoxicosis outbreak characterized by 317 cases of acute hepatic failure and subsequent 125 deaths was reported [17, 35, 36]. The Kenyan Ministry of Health worked collaboratively with experts from the US Center for Disease Control and Prevention to assess risk factors associated with the outbreak [18, 21]. While the cases were inhabitants of Makueni, Kitui, and Machakos Counties, inhabitants of Makueni County were the most affected, accounting for almost 50% of the cases [20, 37]. Consumption of AF-contaminated maize was linked to aflatoxicosis [21, 36].

**Fig. 1:**
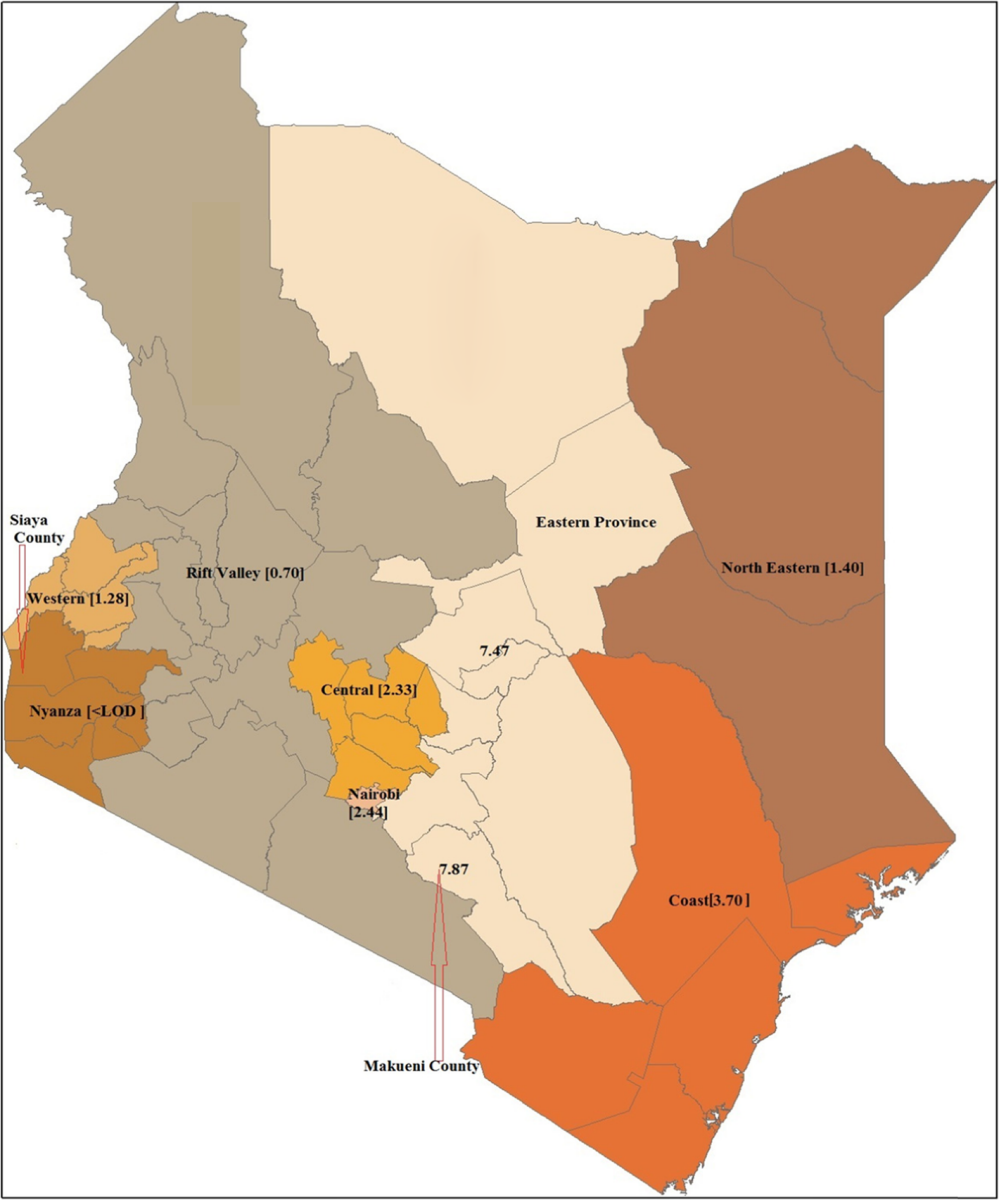
Regional variation of aflatoxin exposure by former provinces, Kenya 2007

In a serological survey to evaluate regional variation of AF exposure in Kenya, 78% of archived serum samples had detectable AF levels [25]. The highest AF exposure was recorded in the former Eastern Province with a median of 7.87 pg/mg AF albumin adducts, while in the former Nyanza Province, the AF albumin adducts were below the limit of detection (< LOD) in human serum samples evaluated. The AF levels were much lower in all the other five provinces. The regional variation of AF exposure levels as reported by Yard et al. [25] is exhibited in Fig. 1 created using vector map outlines from Vemaps.com [38].

#### Sample size determination

According to the Kenya Population census of 2009, Siaya County had a population of 842,304 while Makueni County had 884,527 people [39]. The sample size was determined using Krejcie and Morgan’s methodology [40].

$$ s={X}^2 NP\left[1-P\right]\div {d}^2\left[N-1\right]+{X}^2P\left[1-P\right] $$

where *s* is the required sample size; *X*^2^_=_ 3.841, which is the table value of chi-square for 1 degree of freedom at 95% confidence interval; *N* is the population size; *P* is the population proportion which is assumed to be 0.50 since this provides maximum sample size; and *d* is the degree of accuracy expressed as a proportion (0.05).

According to Krejcie and Morgan’s formula, the estimated sample size was determined to be 393 schoolchildren per county [40]. The estimated total number of participants required to achieve the study objectives was 786 across different schools from 2 counties.

#### Sampling

Makueni is confirmed to have the highest prevalence of AF exposure among Kenya’s 47 counties spread across former 8 provinces. In contrast, Siaya which is considered to be a low exposure region has had few studies conducted in the region. Siaya and Makueni Counties were chosen based on AF exposure levels reported on published literature [20, 25, 37]. Schools were randomly selected per constituency at the county level as long as the school did not have feeding programs and were located at least 3 km from a shopping center to limit the possibility of consuming market or store-bought maize products. In Siaya County, schools allow a 1-h lunch break where students go home for lunch and come back for afternoon classes. In Makueni, however, students bring lunch from home in the morning and have half-hour lunch break, before resuming classes in the afternoon. Participants enrolled in the study were randomly selected using the Kish Grid Method to avoid selection bias [41].

#### Human participants

School administrations were requested to convene parent meetings at a convenient time and location for participants. Community leaders including local area chiefs and members of the county assembly were invited to the meetings. The chairperson of the Parent Teachers Association was in attendance and presided over the meetings. The study purpose and significance were explained to school officials, teachers, support staff, and parents. The participants were given ample opportunity to ask questions during the meeting, before subject recruitment, and during the study to ensure full disclosure of study components. Informed consent forms were explained to parents in their local dialect, and all their questions were addressed before being asked to provide consent and fill out questionnaires.

The five-part questionnaire administered collected basic information including participants’ age, sex, weight, height, and mid-upper arm circumference; sociodemographic factors including marital status, education levels, living conditions, occupations of both the respondent and their spouse, household income levels, and information on home ownership and living conditions. Participants also reported farming practices which entail whether they grow or buy maize for household use, use of pesticides and/or fertilizers during farming, extent of AF knowledge, and how each household stores maize and other food supplies after harvest. In addition, the questionnaire collected information on dietary intake of different food groups, number of meals in a day, food choice, and nutrition practices. Information collected from questionnaires provided sufficient information to enable analysis of socioeconomic status and respective poverty levels. Moreover, these possible confounding factors can be easily controlled in statistical analyses.

**Fig. 2:**
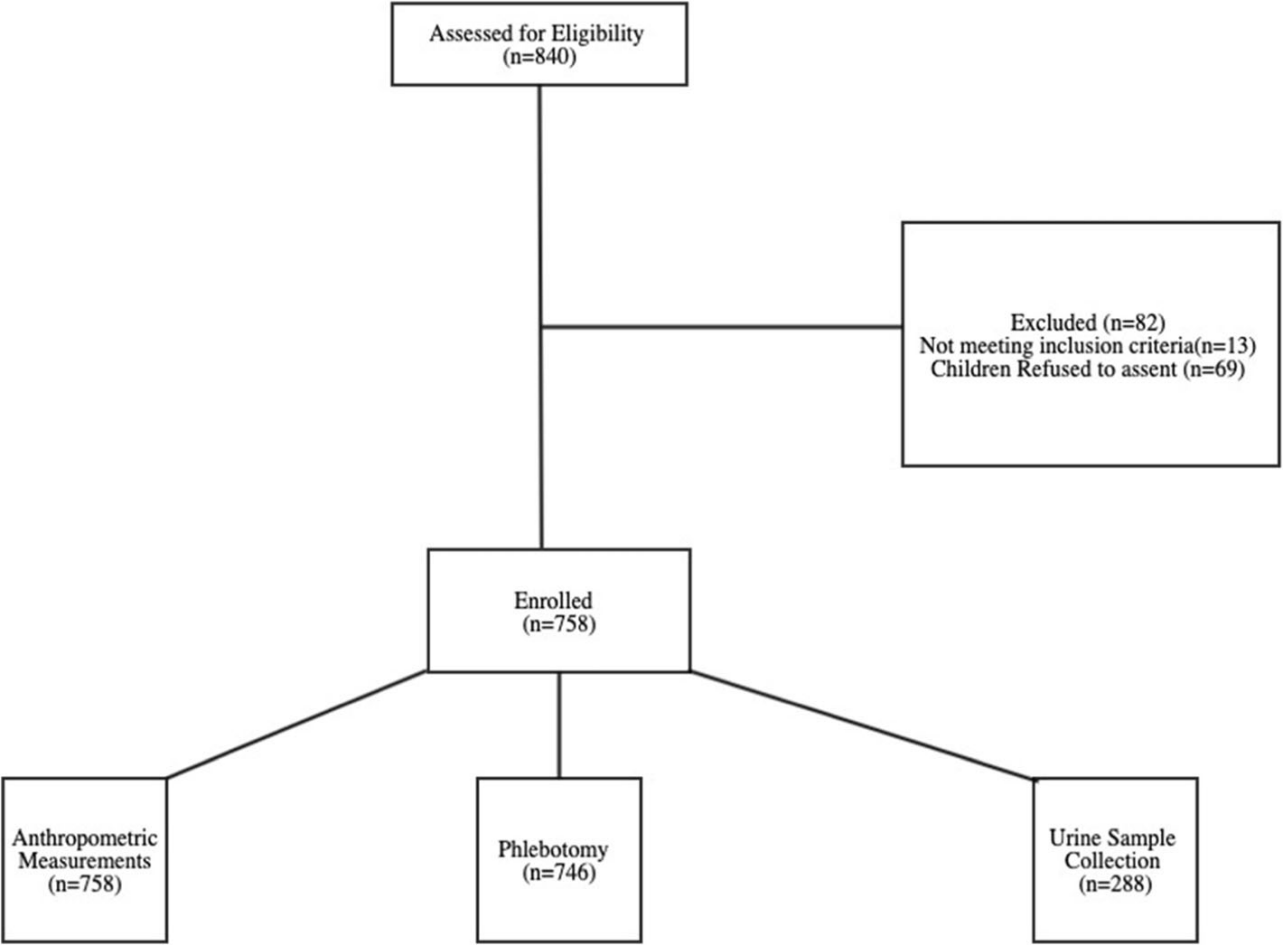
Study participants’ recruitment

After the meeting at various schools, the researchers crosschecked all consent forms and questionnaires for accuracies. In cases where consent was not clear, the individual parent was contacted to confirm their decision. In addition, parents were required to provide additional consent by checking YES or NO on the questionnaire form to allow international shipping of samples, use of media files, storage of biospecimens, and publication of anonymized data summaries. Parents were also asked to provide about 150 g of household maize flour or kernels for AF measurements of household food. Only healthy children between the ages of 6 and 12 with no current active medication were enrolled in the study. After obtaining assent, the study personnel obtained anthropometric measurements including height, weight, and mid-upper arm circumference; 6–8 ml of venous blood; and 15 ml of urine samples. Collection of urine samples was discontinued due to budget constraints and the labor-intensive nature of collecting urine from children. Figure 2 shows a flow diagram showing the study participants’ recruitment and enrollment.

#### Laboratory and data analysis planned

Dietary exposure to AFs can be estimated by quantifying contamination levels in food samples, estimates from dietary intake surveys, and food frequency questionnaires. However, the heterogenous nature of AF contamination in food products does not provide accurate measurement of personal exposures. Therefore, in addition to estimating AF contamination in foods, combinations of AF biomarkers either in blood serum, plasma, tissues, or urine are recommended for studies to determine actual exposure in human sub-populations [42, 43]. Once ingested, AFs undergo metabolism and their metabolites are known to irreversibly bind to protein albumin to form AFB_1_-lysine adducts which are validated biomarkers of AF exposures [44, 45]. Laboratory analyses of this study are currently ongoing, and the study timeline is summarized in Table 1. In addition to quantification of AFB_1_-lysine adducts in serum samples and total AFs in household maize products, plans are underway to quantify other AF metabolites in urine using High Pressure Liquid Chromatography with Fluorescence Detection [44, 45]. STATA15 (College Station, TX), SAS v9.4 (Cary, NC), and R (Vienna, Austria) will be used in adjusting for socioeconomic factors and other possible confounding variables to model data that is likely to associate AF exposure to adverse health outcomes.

**Table 1:**
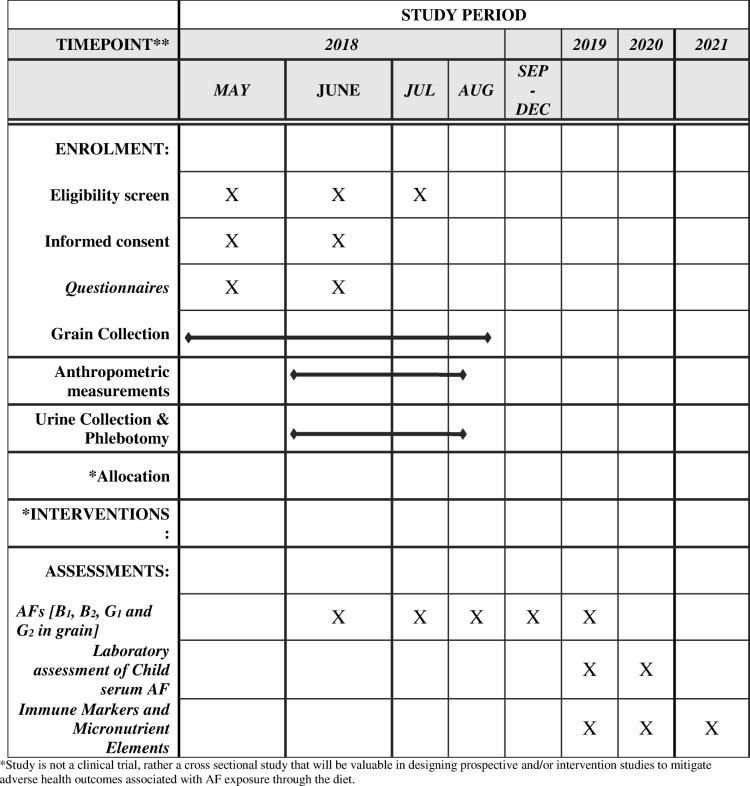
Study timeline

## Discussion

This is the first study designed to establish a baseline AF exposure in children between the ages of 6 and 12 years. To date, studies to establish AF exposure have been completed in populations of children below the age of 5 years, adults between the ages of 18 and 65 years, vulnerable populations of pregnant women, and immunocompromised groups including individuals suffering from HIV/AIDS and hepatitis B virus [6, 12, 46, 47]. Moreover, these studies tend to address specific adverse health outcomes including immune suppression, micronutrient levels, possible growth impairment, and risks of carcinogenesis without controlling for related outcomes. For instance, studies aimed to assess the effect of AF exposure on children’s growth in Benin and Togo prioritized possible growth impairments and micronutrient levels in blood plasma while immune markers were not evaluated [48, 49]. Conversely, a study in Ghana prioritized evaluation of immune suppression as it relates to AF exposure while neither micronutrient markers nor growth indicators were considered [50]. Studies in farm animals and human sub-populations have reported downstream effects associated with AF exposure, malnutrition, immune suppression, vulnerability to infectious diseases, and consequently growth impairments [10, 12, 51]. Intervention studies such as use of enterosorbents like Novasil Clay to bind AF adducts in human blood have been evaluated for safety, palatability, and efficacy [52–55]. In a phase II clinical trial, 180 adults aged 18 to 58 years at risk of aflatoxicosis who received Novasil Clay capsules significantly had lower AFB_1_-lysine adducts in both blood and urine samples in a dose-dependent manner [55]. Other interventions involving use of green tea polyphenols [56] and chlorophyllin [57] have been shown to reduce AF biomarkers and may also form basis for future prospective studies. The current study protocol provides a multifaceted approach in addressing adverse health outcomes linked to dietary exposure to AFs while controlling for social, economic, and demographic factors. While the mechanism by which AFs contribute to adverse health outcomes is currently unknown, it is suspected to be biological [58].

Given the multifaceted aspects of this study, lessons that may be valuable to other scientists seeking to undertake research projects in low- or middle-income countries are outlined herein. First, plan the study at least 2 to 3 years in advance before actual fieldwork to give you and your team sufficient time to prepare, apply for ethics justification, follow up different requirements, and obtain additional permissions required to conduct field studies involving human subjects. Our study’s ethical approval was prolonged because it involved children.

In addition, travel to your country of interest to build social capital. Moreover, foreign scientists are likely to be more successful if they work collaboratively with local scientists in low- and middle-income countries. It is also important to familiarize oneself with myths and beliefs in a community as blood draw is a contentious issue in some communities. In our study populations, a common belief that blood products can used for financial profit by researchers was prohibitive. Moreover, study administrators must make it clear to participants that their samples will be shipped for analysis to a different location and obtain consent for shipment which must be approved a priori by the ethics committee. The paperwork associated with additional approvals for sample shipment, export, and import can be extensive and thorough. Last but not least, remuneration of field workers can exponentiate overhead costs.

### Study limitations

The study administrators are not ethically allowed to contact participants in the future, and thus, study results cannot be used to analyze behavior change or determine cause and effect. Based on results from this study, a prospective cohort study will be valuable in further studies aimed at explaining adverse health effects associated with AF exposure.

We expect AF exposures to be high in May, June, and July because it is rainy and humid, and most farmers have a surplus of harvested grains from the earlier season which are highly susceptible to AF contamination. Nonetheless, this is not guaranteed as factors such as community education programs to promote AF awareness, diet diversification, and food security in some regions where study participants were recruited may be associated with decreased AF exposures.

## Conclusions

This paper describes a protocol of the first school-based randomized cross-sectional study aimed at assessing health effects associated with exposure to AFs through the diet. Whether or not a relationship is found between AF exposure and adverse health outcomes, the results can be used to prioritize AF control efforts not only in Kenya but also in other developing countries.

The strength of the study is its multifaceted approach in assessing health effects in children exposed to AFs through the diet. In addition, recruiting subjects from both a high and a low exposure region is instrumental in elucidating the role of AF exposure specifically on micronutrient deficiency, immune suppression, and possible growth impairments.

In conclusion, exposure to AFs and other mycotoxins in low- and middle-income countries has been shown to contribute to immune suppression and growth impairment. Mycotoxins are also suspected to interfere with vaccine efficiency making children more vulnerable to increased risk of infectious diseases. Pediatricians and clinicians in developing nations should pay attention to the role of not only AFs but also other environmental factors that influence health in their practice to better serve their populations.

## Data Availability

Data sharing is not applicable to this article as no datasets were generated or analyzed during the current study. Only study investigators will have access to the final dataset.
